# First-in-human SPECT/CT imaging of [^212^Pb]Pb-VMT-α-NET in a patient with metastatic neuroendocrine tumor

**DOI:** 10.1007/s00259-023-06529-1

**Published:** 2023-11-22

**Authors:** Enrico Michler, David Kästner, Claudia Brogsitter, Marc Pretze, Holger Hartmann, Robert Freudenberg, Michael K. Schultz, Jörg Kotzerke

**Affiliations:** 1 Department of Nuclear Medicine, University Hospital Carl Gustav Carus, Technische Universität Dresden, Fetscherstraße 74, 01307 Dresden, Germany; 2 Perspective Therapeutics, Inc., Coralville, IA USA; 3 Faculty of Medicine Carl Gustav Carus, Technische Universität Dresden, Dresden, Germany

^212^Pb is a promising radionuclide for targeted alpha particle therapy for cancer. Ongoing preclinical and clinical studies are investigating the potential of ^212^Pb-labeled peptides and antibodies [1–5]. PSC-PEG_2_-TOC (VMT-α-NET) is a novel somatostatin receptor subtype 2 (SSTR2) targeting peptide for the treatment of neuroendocrine tumors (NET) that shows rapid tumor accumulation, high tumor retention, and fast renal excretion with the potential for low nephrotoxicity [6, 7].

Here, we present the case of a 75-year-old woman with an advanced G2 NET of unknown primary with liver metastases who was heavily pretreated with somatostatin analogs, various chemotherapies, multiple cycles of [^177^Lu]Lu-DOTA-TATE and [^225^Ac]Ac-DOTA-TATE, and radioembolization over 7 years. The patient received 90 MBq of [^212^Pb]Pb-VMT-α-NET intravenously. Whole-body scintigraphy and SPECT/CT acquisitions were performed 2, 5, and 19 h after injection on a Symbia Intevo 6 (Siemens Healthineers) using high-energy collimators. Images were obtained by detection of the characteristic X-ray emissions of ^212^Pb using an energy window at 79 keV (40% width). The whole-body scan speed was 8 cm/min, and SPECT/CT scans were acquired with 120 projections (60 per detector, 30 s per projection) over a non-circular 360° orbit. The SPECT/CT images showed a high accumulation of [^212^Pb]Pb-VMT-α-NET in liver metastases in segments III and IV, consistent with the previously acquired [^68^Ga]Ga-DOTA-TATE PET/CT. High tumor retention can be observed in the planar and SPECT/CT images over time. The planar images showed a high level of background noise due to down-scatter and septal penetration of high-energy photon emissions from ^212^Pb daughter nuclides (e.g., 2.6 MeV from ^208^Tl). Due to the short half-life of ^212^Pb (10.6 h), the images acquired after 19 h showed a relatively high level of image noise due to the low count statistics. The patient showed no early or acute adverse events.

These are the first clinical post-treatment scintigraphic images of [^212^Pb]Pb-VMT-α-NET and additionally the first-in-human SPECT/CT images of ^212^Pb.
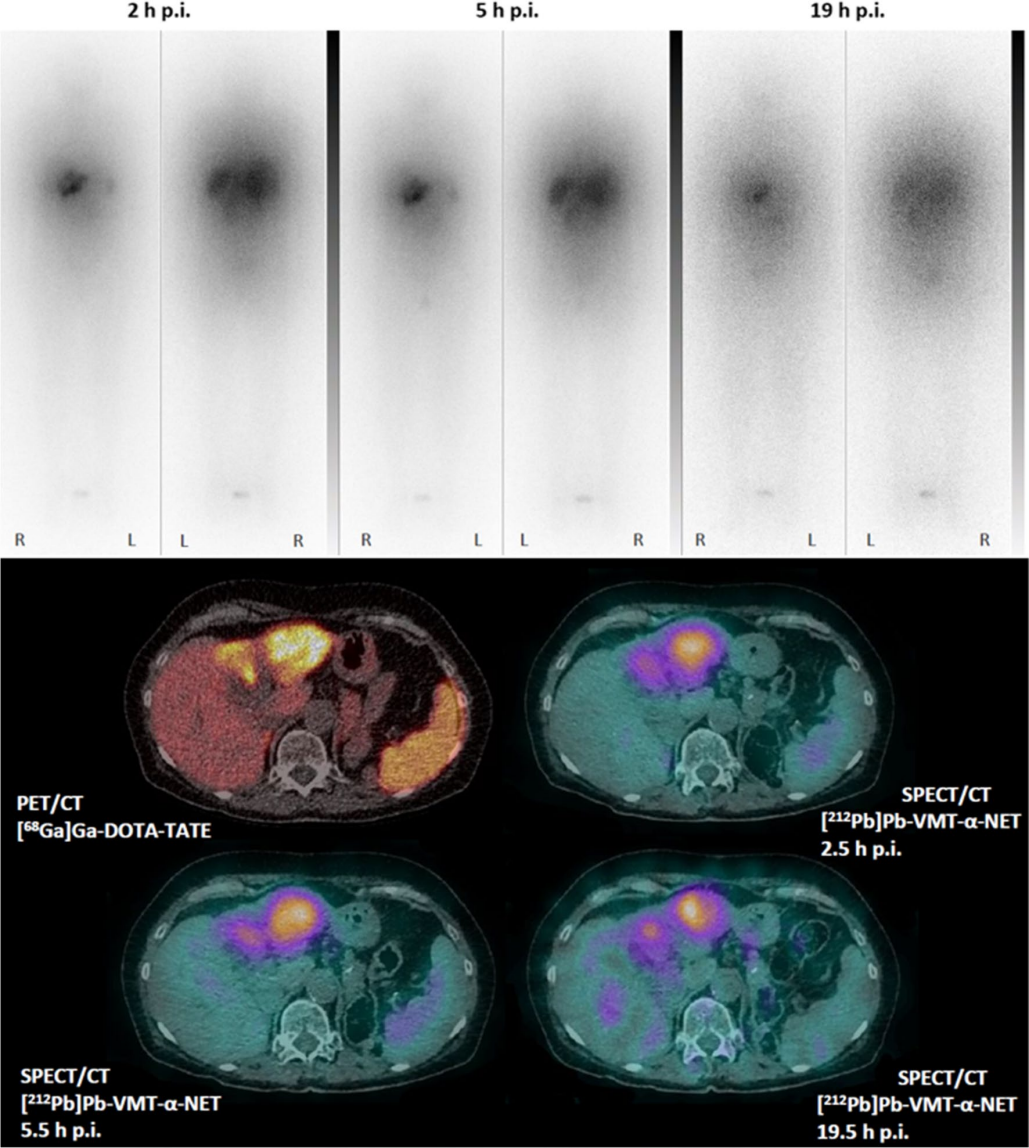


## Data Availability

The data of this study are available on reasonable request.
